# COVID-19 vaccine hesitancy in rural South Africa: Deepening understanding to increase uptake and access

**DOI:** 10.7189/jogh.12.05013

**Published:** 2022-05-14

**Authors:** Kathleen Kahn, Audrey Pettifor, Palesa Mataboge, Nicole K Kelly, Duduzile P Mashinini, Harish Nair, Harry Campbell, Cheryl Cohen, F Xavier Gómez-Olivé, Stephen Tollman

**Affiliations:** 1 MRC/Wits Rural Public Health and Health Transitions Research Unit, School of Public Health, Faculty of Health Sciences, University of the Witwatersrand, Johannesburg, South Africa; 2 Department of Epidemiology, Gillings School of Global Public Health, University of North Carolina at Chapel Hill, Chapel Hill, North Carolina, USA; 3 Carolina Population Center, University of North Carolina at Chapel Hill, Chapel Hill, North Carolina, USA; 4 College of Medicine and Veterinary Medicine, University of Edinburgh, Edinburgh, UK; 5 Centre for Respiratory Diseases and Meningitis, National Institute for Communicable Diseases of the National Health Laboratory Service, Johannesburg, South Africa; 6 School of Public Health, Faculty of Health Sciences, University of Witwatersrand, Johannesburg, South Africa

## Abstract

**Background:**

To date, COVID-19 vaccine coverage in the African region falls far too short of global goals. Increasing vaccination rates requires understanding barriers to vaccination so that effective interventions that sensitively and effectively address barriers to vaccination can be implemented.

**Methods:**

To assess COVID-19 vaccination levels and identify major barriers to vaccine uptake we conducted a population-based, cross-sectional survey among 1662 adults 18 and older from August 25 to October 29 2021 in the Agincourt Health and Socio-Demographic Surveillance System (AHDSS) area, Mpumalanga, South Africa.

**Results:**

Half of participants reported receiving a COVID-19 vaccine (50.4%) with 41.1% being fully vaccinated and 9.3% being partially vaccinated; 49.6% were unvaccinated. More women than men were vaccinated (55.5% vs 42.8%, *P* < 0.001), and older age groups were more likely to be vaccinated than younger age groups (*P* < 0.001). Among the unvaccinated, 69.0% planned to get vaccinated as soon as possible, while 14.7% reported definitely not wanting the vaccine. Major barriers to vaccination included lacking information on eligibility (12.3%) or where to get vaccinated (13.0%), concerns about side effects (12.5%), and inconvenient hours and locations for vaccination (11.0%). Confidence in the safety and efficacy of COVID-19 vaccines was higher among those vaccinated than unvaccinated (75.3% vs 51.2%, 75.8% vs 51.0%, both *P* < 0.001, respectively).

**Conclusions:**

Increasing vaccination in South Africa beyond current levels will require a concerted effort to address concerns around vaccine safety and increase confidence in vaccine efficacy. Clarifying eligibility and ensuring access to vaccines at times and places that are convenient to younger populations, men, and other vulnerable groups is necessary.

The emergence of the Omicron variant in late 2021 and early 2022 has reinforced the effects of COVID-19 vaccine inequity on the pandemic. As many Western countries such as the United Kingdom and United States responded to the identification of the highly transmissible Omicron variant by closing borders to citizens of southern African countries [1,2], scientists have questioned the effectiveness of border restrictions, highlighted the knock-on effect on economies and households, and rather emphasized the need to reduce the huge disparity in vaccine access in low- and middle-income countries (LMICs) as the sustainable solution [3,4].

Vaccination rates in LMICs, particularly in Africa, continue to fall short of global goals [5,6]. Although it is clear that access to vaccines is a key determinant of vaccine coverage, it is also affected by vaccine hesitancy and implementation challenges. However, better data are needed to determine the barriers to vaccination to assist with tailoring interventions that will improve vaccine uptake in the African region. Research in South Africa has shown that there has been a general shift towards the acceptance of vaccines since the start of the COVID-19 pandemic; however, approximately 29% of adults reported being unwilling to be vaccinated in May/April of 2021 [7]. Some primary concerns that have fueled COVID-19 vaccine hesitancy in South Africa and the African region include concerns about safety, which in part is due to the rapid pace of vaccine development [7,8].

Growing anti-vaccine sentiment in Africa, especially in South Africa, has recently garnered global attention [9]. Even before the COVID-19 pandemic, vaccine hesitancy in South Africa was implicated in several vaccine-preventable disease outbreaks, including a measles outbreak involving 18 311 cases between 2009 and 2010 [10]. Expectedly, COVID-19 vaccination efforts in the country have again brought this issue to the forefront. For instance, in their evaluation of the COVID-19 vaccine rollout for health care workers in South Africa in early 2021, Wiysonge et al. revealed high levels of vaccine hesitancy at 41% among health care workers in Cape Town, those who would be charged with administering vaccines [11]. In their evaluation of vaccine hesitancy surveys conducted in South Africa prior to and during the start of COVID-19 vaccine rollout, Cooper et al. acknowledge the fluctuating COVID-19 vaccine acceptance [9], reiterating the urgent need for studies that would deepen the understanding of vaccine hesitancy in South Africa and inform interventions for increasing uptake.

## METHODS

Aiming to cover this information gap, we conducted a population-based, cross-sectional telephonic survey among individuals ages 18 and older from August 25 to October 29, 2021, in the Agincourt Health and Socio-Demographic Surveillance System (AHDSS) area located in Mpumalanga province, northeast South Africa, adjacent to southern Mozambique. The rural setting is characterized by high levels of poverty, unemployment, and circular labour migration [12]. From May 2021, COVID-19 vaccines became available to adults aged 60 and older, and from September 1, 2021, South Africans 18 and older were eligible to receive the COVID-19 vaccine (Pfizer and Jansen are available). 1664 adults completed the telephonic interview, and 1662 (99.9%) completed the vaccine module and were included in this analysis. This survey was part of an ongoing panel, comprising a cohort of individuals measuring the impact of COVID-19 on economic, behavioural, and mental health outcomes in the AHDSS. The original sample was selected in August 2020 and included 2300 households (one individual per household was sampled). The sample was selected to be representative of the population and to ensure a sufficient sample size to generate precision around estimates by gender and age groups.

We measured factors that have been shown to impact vaccine uptake. Based on the theoretical model of increasing vaccination put forward by Brewer et al. and adapted by the World Health Organization (WHO) working group, we measured items in the constructs that shape vaccination uptake [13,14]: 1) what people think and feel – confidence in the vaccine safety and efficacy; 2) social processes – family and social support for vaccination; 3) motivation – intention to get vaccinated and 4) practical issues – knowledge of where the vaccine is available, ease of access, etc. The survey asked individuals about demographics, vaccine uptake (whether they had received the COVID-19 vaccine, which vaccine, when they received it, how many doses), social processes (disclosure of vaccine status to others and encouraging others to be vaccinated); the proportion of family and friends that are vaccinated or intend to be vaccinated; trusted sources of vaccine information; support from local and national leaders around vaccination; what people think and feel (confidence in vaccine safety and efficacy, reasons for vaccination or non-vaccination); practical issues (ease of access to the vaccine and knowledge of where to get the vaccine). Data were weighted by age and gender to be representative of the AHDSS from 2020 (total AHDSS eligible population = 34 582). Specifically, inverse probability of selection weights were applied to each stratum of gender (female/male) and age (age groups: 18-29, 30-39, 40-49, 50-59, 60-69, 70-79). Weighted descriptive results are presented below (counts, percentages, and 95% confidence intervals (CIs)) and χ^2^ tests were conducted to preliminarily assess differences between groups. All statistical testing was two-sided with an alpha of 0.05; analyses were conducted using Stata version 16.1 (StataCorp LLC, College City Texas, USA). Verbal informed consent was obtained from all participants and ethical approval was obtained from the Human Research Ethics Committee at the University of the Witwatersrand and the Institutional Review Board of the University of North Carolina.

## RESULTS

Half of the participants reported receiving a COVID-19 vaccine (50.4%, 95% CI = 47.9-52.9) with 41.1% (95% CI = 38.7-43.5) being fully vaccinated and 9.3% (95% CI = 8.0-10.7) partially vaccinated, while 49.6% (95% CI = 47.1-52.1) were not vaccinated. More women than men were vaccinated (55.5% of women vs 42.8% of men, *P* < 0.001), and older age groups were more likely to be vaccinated than younger age groups (*P* < 0.001) (**Table 1**). Of those vaccinated, 68.6% (95% CI = 65.4-71.7) reported having been vaccinated at a community venue such as a church or school, and 96.4% (95% CI = 94.8-97.4) reported that the main reason they accepted vaccination was to protect their health and that of their families, friends, and communities. When asked if they had disclosed their vaccination status to anyone, 95.2% (95% CI = 93.6-96.5) reported that they had. Among vaccinated individuals, 87.3% (95% CI = 85.0-89.4) anticipated that most of their family and friends would get a COVID-19 vaccine compared to only 70.5% (95% CI = 67.0-73.7) of unvaccinated individuals (*P* < 0.001). 96.5% (95% CI = 95.1-97.5) of vaccinated participants and 85.9% (95% CI = 83.1-88.3) of unvaccinated participants personally knew someone who had been vaccinated (*P* < 0.001).

**Table 1:** Demographic characteristics among adults 18 and older in the Agincourt Health and Socio-Demographic Surveillance System (AHDSS) area, Mpumalanga, South Africa. ^*†^

			Unvaccinated (n = 17 158)		Vaccinated (n = 17 424)	
	**n**	**95% CI**	**n**	**% (CI)**	**n**	**95% CI**
**Age group**						
18-29	8981	52.3 (48.7-55.9)	3730	21.4 (18.5-24.6)	12 711	36.8 (34.3-39.3)
30-39	4123	24.0 (21.2-27.1)	3228	18.5 (16.1-21.3)	7351	21.3 (19.4-23.3)
40-49	2148	12.5 (10.5-14.8)	3428	19.7 (17.3-22.3)	5576	16.1 (14.5-17.8)
50-59	1228	7.2 (5.6-9.1)	3055	17.5 (15.2-20.1)	4283	12.4 (10.9-14.0)
60-69	446	2.6 (1.7-3.8)	2565	14.7 (12.6-17.1)	3011	8.7 (7.5-10.1)
70+	232	1.4 (0.8-2.3)	1418	8.1 (6.6-10.0)	1650	4.8 (3.9-5.8)
**Gender**						
Female	9198	53.6 (49.9-57.2)	11 475	65.9 (62.5-69.1)	20 673	59.8 (57.3-62.2)
Male	7960	46.4 (42.8-50.1)	5949	34.1 (30.9-37.5)	13 909	40.2 (37.8-42.7)
**Currently employed**						
Yes	5175	30.2 (26.9-33.6)	5823	33.4 (30.3-36.7)	10 998	31.8 (29.5-34.2)
No	11 733	68.4 (64.9-71.7)	11 439	65.7 (62.4-68.8)	23 172	67.0 (64.6-69.3)
Missing	251	1.5 (0.8-2.6)	161	0.9 (0.5-1.7)	412	1.2 (0.8-1.8)
**Food insecure**						
Yes	5469	31.9 (28.6-35.3)	5704	32.7 (29.7-35.9)	11 172	32.3 (30.1-34.6)
No	11 479	66.9 (63.4-70.2)	11 537	66.2 (63.0-69.3)	23 016	66.6 (64.2-68.8)
Missing	211	1.2 (0.6-2.4)	183	1.1 (0.5-2.0)	394	1.1 (0.7-1.8)

CI – Confidence interval

*August 25 to October 29, 2021, stratified by COVID-19 vaccination status and weighted to be representative of the AHDSS by age and gender.

† All counts (n), percentages (%), and 95% CIs are weighted values, including those for missing data.

Of the 49.6% who had not received any dose of a COVID-19 vaccine, when asked if they would get vaccinated, 69.0% (95% CI = 65.5-72.3) said they would get vaccinated as soon as they could, 8.0% (95% CI = 6.2-10.2) said they would wait a while, 7.4% (95% CI = 5.7-9.6) reported being unsure, and 14.7% (95% CI = 12.3-17.5) said they would definitely not get vaccinated. When unvaccinated participants were asked why they had not been vaccinated, the most common reasons were lack of information (36.6%, 95% CI = 33.1-40.2), such as not knowing if they were eligible and not knowing where to go (**Table 2**). This was followed by COVID-19 vaccine hesitancy (19.7%, 95% CI = 16.9-22.8), such as concerns about side effects, and health care access issues (14.3%, 95% CI = 11.9-17.1), such as vaccination sites being open at inconvenient hours, long wait times, or distance from the site. The hesitancy toward vaccines in general (8.4%, 95% CI = 6.6-10.7) and possible contraindications to the vaccine (6.2%, 95% CI = 4.7-8.0) were other relevant factors contributing to not receiving the vaccine. Among the 14.7% who said they would definitely not get the COVID-19 vaccine, there was a clear age gradient from youngest to oldest, 50.9% were 18-29-year-olds, 21.3% were 30-39-year-olds, 12.5% were 40-49-year-olds, 8.8% were 50-59-year-olds, 4.1% were 60-69-year-olds, and 2.5% were 70+ year-olds; there was no difference by gender. When examining the perceived risk of COVID-19 among the 14.7% who said they had no plans to be vaccinated compared to those who said they did plan to get vaccinated, 27.9% (95% CI = 20.0-37.3) of those who did not plan to get vaccinated thought it was moderately/extremely likely they would get seriously ill if they got COVID-19 compared to 49.2% (95% CI = 45.3-53.2), (*P* < 0.001) of those who planned to get vaccinated.

**Table 2:** Primary reason for not getting a COVID-19 vaccine among adults in Mpumalanga, South Africa, stratified by gender*

	Male (n = 7960)		Female (n = 9198)		Total (n = 17 158)	
**Main reason for not getting vaccinated**	**n**	**95% CI**	**n**	**95% CI**	**N**	**95% CI**
**Communication/outreach issues:**	**2638**	**33.1 (28.1-38.7)**	**3638**	**39.5 (35.0-44.3)**	**6276**	**36.6 (33.1-40.2)**
Didn’t know where to go	1134	14.2 (10.8-18.6)	1103	12.0 (9.2-15.5)	2236	13.0 (10.8-15.7)
Didn’t know I was eligible	912	11.5 (8.3-15.6)	1193	13.0 (10.1-16.5)	2106	12.3 (10.1-14.9)
Not eligible	592	7.4 (4.9-11.1)	1,271	13.8 (10.8-17.5)	1863	10.9 (8.8-13.3)
Didn’t know about the vaccine/it was available	0	0	71	0.8 (0.3-2.0)	71	0.4 (0.2-1.1)
**COVID-19 vaccine hesitancy:**	**1860**	**23.4 (18.9-28.5)**	**1523**	**16.6 (13.3-20.4)**	**3383**	**19.7 (16.9-22.8)**
Concerned about side effects	1213	15.2 (11.6-19.8)	930	10.1 (7.6-13.3)	2143	12.5 (10.3-15.1)
Concerned vaccine will cause spiritual harm	307	3.9 (2.2-6.7)	199	2.2 (1.1-4.3)	506	3.0 (1.9-4.5)
Not ready yet/wants more info/not sure	155	1.9 (0.9-4.3)	238	2.6 (1.4-4.8)	393	2.3 (1.4-3.7)
Don’t think vaccine will work	161	2.0 (0.9-4.5)	95	1.0 (0.4-2.5)	255	1.5 (0.8-2.7)
Scared of the vaccine	0	0	61	0.7 (0.2-2.1)	61	0.4 (0.1-1.1)
Don’t understand why the vaccine is needed	24	0.3 (0.0-2.1)	0	0	24	0.1 (0.0-1.0)
**Structural & supply-side barriers to health care:**	**1211**	**15.2 (11.6-19.7)**	**1241**	**13.5 (10.5-17.1)**	**2452**	**14.3 (11.9-17.1)**
Vaccination site open at inconvenient hours/long wait times	991	12.4 (9.2-16.7)	890	9.7 (7.2-12.9)	1881	11.0 (8.9-13.5)
Too long/expensive to travel to vaccination site	95	1.2 (0.4-3.2)	236	2.6 (1.4-4.7)	330	1.9 (1.1-3.2)
Registration issues	106	1.3 (0.5-3.4)	26	0.3 (0.0-2.0)	132	0.8 (0.3-1.8)
Clinic ran out of vaccines	19	0.2 (0.0-1.7)	89	1.0 (0.4-2.6)	108	0.6 (0.3-1.5)
**General vaccine hesitancy:**	**775**	**9.7 (6.9-13.6)**	**668**	**7.3 (5.1-10.2)**	**1443**	**8.4 (6.6-10.7)**
Don’t get vaccines generally	471	5.9 (3.7-9.2)	452	4.9 (3.2-7.5)	923	5.4 (3.9-7.3)
Don’t like needles	275	3.5 (1.9-6.2)	189	2.1 (1.1-4.0)	464	2.7 (1.7-4.2)
People in my community do not get vaccines	29	0.4 (0.1-2.6)	26	0.3 (0.0-2.0)	56	0.3 (0.1-1.3)
**Possible contraindications:**	**194**	**2.4 (1.2-4.9)**	**862**	**9.4 (7.0-12.4)**	**1056**	**6.2 (4.7-8.0)**
Currently sick	145	1.8 (0.8-4.1)	478	5.2 (3.6-7.5)	624	3.6 (2.6-5.1)
Pregnant/breastfeeding	0	0	173	1.9 (0.9-3.9)	173	1.0 (0.5-2.1)
Allergic to vaccines	49	0.6 (0.1-2.5)	69	0.7 (0.3-2.0)	117	0.7 (0.3-1.6)
Concerned about concomitant medications	0	0	101	1.1 (0.5-2.4)	101	0.6 (0.3-1.3)
Concerned about comorbidity	0	0	42	0.5 (0.1-1.9)	42	0.2 (0.1-1.0)
**Lack of concern about COVID-19:**	**130**	**1.6 (0.7-3.9)**	**42**	**0.5 (0.1-1.9)**	**172**	**1.0 (0.5-2.1)**
Not concerned about getting ill from COVID-19	106	1.3 (0.5-3.5)	16	0.2 (0.0-1.2)	122	0.7 (0.3-1.7)
COVID-19 is not as serious as people say	24	0.3 (0.0-2.1)	26	0.3 (0.0-2.0)	50	0.3 (0.1-1.2)
**Other:**	**1151**	**14.5 (11.0-18.8)**	**1174**	**12.8 (9.9-16.3)**	**2325**	**13.5 (11.3-16.2)**
Unknown “other” reason	1009	12.7 (9.4-16.8)	1062	11.5 (8.8-14.9)	2071	12.1 (9.9-14.6)
Other	142	1.8 (0.8-3.9)	111	1.2 (0.5-2.7)	253	1.5 (0.8-2.6)
Missing	0	0	52	0.6 (0.2-1.8)	52	0.3 (0.1-0.9)

CI – Confidence interval

*All counts (n), percentages (%), and 95% CIs are weighted values, including those for missing data.

As expected, confidence in COVID-19 vaccine safety was higher among those vaccinated with 75.3% (95% CI = 72.3-78.0) being somewhat or very confident the COVID-19 vaccine is safe vs only 51.2% (95% CI = 47.6-54.9) of unvaccinated individuals (*P* < 0.001). Similarly, 75.8% (95% CI = 72.9-78.5) of the vaccinated were somewhat or very confident that the COVID-19 vaccine is effective vs 51.0% (95% CI = 47.3-54.6) of the unvaccinated (*P* < 0.001). The individuals most trusted for providing accurate information on COVID-19 vaccines were reported to be doctors (58.0%, 95% CI = 55.5-60.4) and nurses (49.1%, 95% CI = 46.6-51.5) for both vaccinated and unvaccinated individuals (**Table 3**). Government officials were endorsed by 15.9% (95% CI = 14.2-17.8), and family and friends were trusted by only 6.1% (95% CI = 5.0-7.5). Most individuals obtained information on COVID-19 vaccines from TV (59.2%, 95% CI = 56.7-61.6) and radio (46.1%, 95% CI = 43.7-48.6) followed by social media (24.8%, 95% CI = 22.6-27.1). Far fewer obtained information from friends, family, church, school, or websites (all less than or equal to 5.0% each); importantly, for the unvaccinated among the 18-29 age group, 42.4% (95% CI = 37.1-47.9) reported getting information on vaccines from social media.

**Table 3:** Sources of COVID-19 vaccination information, support, and trust among adults in Mpumalanga, South Africa, stratified by vaccination status*

	Unvaccinated (n = 17 158)		Vaccinated (n = 17424)		Total (n = 34 582)	
	**n**	**95% CI**	**n**	**95% CI**	**n**	**95% CI**
**Sources for COVID-19 vaccine information:**						
TV	9852	57.4 (53.8-61.0)	10 604	60.9 (57.5-64.1)	20 457	59.2 (56.7-61.6)
Radio	7276	42.4 (38.9-46.0)	8668	49.7 (46.4-53.1)	15 944	46.1 (43.7-48.6)
Social media	5171	30.1 (26.8-33.7)	3404	19.5 (16.9-22.5)	8576	24.8 (22.6-27.1)
Community meetings	2403	14.0 (11.7-16.7)	2530	14.5 (12.4-17.0)	4934	14.3 (12.6-16.1)
Healthcare facilities	2424	14.1 (11.8-16.8)	2480	14.2 (12.1-16.6)	4904	14.2 (12.6-15.9)
Family or friends	960	5.6 (4.1-7.5)	822	4.7 (3.5-6.4)	1782	5.2 (4.1-6.4)
Newspapers	275	1.6 (0.9-2.9)	171	1.0 (0.5-1.9)	446	1.3 (0.8-2.0)
**Trusted sources for accurate vaccine information:**						
Doctors	10 284	59.9 (56.3-63.4)	9773	56.1 (52.8-59.4)	20 057	58.0 (55.5-60.4)
Nurses	7859	45.8 (42.2-49.5)	9105	52.3 (48.9-55.6)	16 964	49.1 (46.6-51.5)
Government officials	2351	13.7 (11.4-16.4)	3162	18.1 (15.7-20.9)	5512	15.9 (14.2-17.8)
Community leaders	1288	7.5 (5.8-9.7)	1683	9.7 (7.9-11.8)	2970	8.6 (7.3-10.1)
Celebrities	1051	6.1 (4.6-8.1)	1085	6.2 (4.8-8.1)	2136	6.2 (5.1-7.5)
Family or friends	1066	6.2 (4.7-8.2)	1056	6.1 (4.6-7.9)	2122	6.1 (5.0-7.5)
Traditional healers	286	1.7 (0.9-2.9)	290	1.7 (1.0-2.7)	576	1.7 (1.1-2.4)
**Perceived leader support of COVID-19 vaccines:**						
Traditional leaders	8434	49.2 (45.5-52.8)	10 339	59.3 (56.0-62.6)	18 773	54.3 (51.8-56.7)
Healthcare workers	9118	53.1 (49.5-56.8)	9295	53.3 (50.0-56.7)	18 413	53.2 (50.8-55.7)
Political leaders	6606	38.5 (35.0-42.1)	8119	46.6 (43.3-49.9)	14 725	42.6 (40.2-45.0)
Teachers	5735	33.4 (30.0-37.0)	5927	34.0 (30.9-37.3)	11 662	33.7 (31.4-36.1)
Religious leaders	5481	31.9 (28.6-35.4)	6055	34.8 (31.6-38.0)	11 536	33.4 (31.1-35.7)

CI – Confidence interval

*All counts (n), percentages (%), and 95% confidence intervals (CIs) are weighted values, including those for missing data.

## DISCUSSION

While there has been much debate on the reasons for low vaccine coverage in Africa, clearly the causes are multi-faceted and involve both structural factors, such as vaccine supply and distribution [15], and on-the-ground demand [8,9]. Across Africa, access to vaccines remains an issue, as the majority of countries have less than 10% of adults fully vaccinated [6]. Wealthier nations must continue to provide vaccines to LMICs and to support vaccine distribution, especially in rural, inner-city, and remote locations. While South Africa is an outlier in the region, now having sufficient availability of vaccines, ensuring easy access to those who want them, and addressing concerns around vaccine safety and efficacy are still major challenges. In particular, efforts should prioritize those for whom access is lower and most tenuous, including men, younger populations, labour migrants, undocumented migrants, and the unhoused, to name a few.

Our findings support the Increasing Vaccination Model put forward by the WHO working group on behavioural and social drivers of vaccination [14]. We observed significant differences between vaccinated and unvaccinated individuals in their thoughts and feelings about vaccines, particularly concerning confidence in vaccine safety and efficacy, and also in their risk perception about the severity of illness if they were to become infected with COVID-19. This relationship is also supported by Kollamparabil et al., who highlight the moderating effect risk perception has on COVID-19 vaccine confidence and thus behaviour in the South African context [16]. Social processes seemed less important in this setting, and while unvaccinated individuals knew fewer vaccinated individuals and anticipated fewer friends and family would be vaccinated than vaccinated individuals, both groups reported very high levels of vaccination in their peer groups. In this study population, access was a major contributor to non-vaccination with confusion about where to get vaccinated, eligibility for vaccination, and also factors associated with the ability to access vaccination sites. Interestingly, interventions that directly impact behaviour change such as vaccine mandates, incentives, default appointments, and on-site vaccinations have shown the greatest likelihood of impact in increasing vaccination uptake [14]. In addition, health care provider recommendations for vaccination have been found to be one of the best interventions for increasing vaccine uptake [14]. In our study population, doctors and nurses were the most trusted source for accurate information on vaccines among vaccinated and unvaccinated individuals. In the analysis in early 2021 looking at COVID-19 vaccine hesitancy in South Africa, trust in health care workers did not predict lower vaccine hesitancy thus the authors posit that health care workers have not been used effectively to address misinformation [17]. How trust in health care workers can be best translated into interventions in LMIC settings to address misinformation and increase vaccination coverage is key. Therefore, along with the aforementioned interventions, we recommend a concerted effort by doctors and nurses to address vaccine concerns. We also endorse the continued use of television and radio to inform the public about vaccinations. However, a greater emphasis needs to be put on using these outlets to mitigate confusion about eligibility and vaccination site locations.

The limitations of this research were in the telephonic conducting of interviews, because of which verification of vaccination status was not possible. Further, the survey was done during the roll-out of the COVID-19 vaccine in South Africa, thus vaccine uptake will currently likely be much higher in this setting, given the interest in vaccination among those reporting to be unvaccinated at the time of the survey. Nevertheless, at the time of writing this article, only 40% of adults 18 and older were vaccinated in Mpumalanga [18]. The study site is located in a rural part of South Africa, so the results are mostly generalizable to other rural populations in South Africa and the southern African region. However, vaccine access is much higher in South Africa than in its neighbour countries.

## CONCLUSIONS

Increasing vaccination in South Africa beyond current levels will require a concerted effort to address concerns around vaccine safety and increase confidence in vaccine efficacy. Understanding the local context and barriers to uptake as vaccines are being rolled out is imperative for effective messaging that addresses real concerns about vaccines, engages with hesitancy sensitively and effectively, and makes it clear where and when to obtain them. Further, we recommend continuing to provide vaccines at convenient times (e.g., outside of working hours), through mobile vaccination clinics, in community venues, and in workplaces, which will help to reach those in need (e.g., younger populations, men, and vulnerable populations), particularly as COVID-19 variants of concern continue to sweep through the region.

## References

[1] US embassy SA travel. Available: https://za.usembassy.gov/visas/. Accessed: 22 February 2022.

[2] UK Foreign TravelAvailable: https://www.gov.uk/foreign-travel-advice/south-africa/coronavirus. Accessed: 22 February 2022.

[3] Mendelson M Venter F Moshabela M Gray G Blumberg L de Oliveira T The political theatre of the UK’s travel ban on South Africa. Lancet. 2021;398:2211-3. 10.1016/S0140-6736(21)02752-5 34871546 PMC8641956

[4] Pai M Olatunbosun-Alakija A Vax the world. Science. 2021;374:1031-1031. . 10.1126/science.abn3081 34822275

[5] World Health Organization. Strategy to Achieve Global Covid-19 Vaccination by Mid-2022. Available: https://www.who.int/publications/m/item/strategy-to-achieve-global-covid-19-vaccination-by-mid-2022. Accessed: 22 February.

[6] Our World in DataAvailable: https://ourworldindata.org/covid-vaccinations. Accessed: 22 February 2022.

[7] Burger R, Maughan-Brown B, Kohler T, English R, Tameris M. A Shot in the Arm for South Africa- Increased Openness to Accepting a COVID-19 Vaccine Evidence from NIDS-CRAM Waves 4 and 5. Available: https://cramsurvey.org/wp-content/uploads/2021/07/2.-Burger-R.-Maughan-Brown-M.-Kohler-T.-English-R.-_-Tameris-M.-2021-Increased-openness-to-accepting-a-COVID-19-vaccine-is-a-shot-in-the-arm-for-South-Africa-Evidence-from-the-NIDS-CRAM-Wave-5-Survey.pdf. Accessed: 22 February 2022.

[8] Africa CDC. COVID-19 Vaccine Perceptions: A 15 Country Study. Available: https://africacdc.org/download/covid-19-vaccine-perceptions-a-15-country-study/. Accessed: 22 Fe bruary 2022.

[9] Cooper S van Rooyen H Wiysonge CS COVID-19 vaccine hesitancy in South Africa: how can we maximize uptake of COVID-19 vaccines? Expert Rev Vaccines. 2021;20:921-33. 10.1080/14760584.2021.1949291 34252336

[10] le Roux DM le Roux SM Nuttall JJ Eley BS South African measles outbreak 2009 - 2010 as experienced by a paediatric hospital. S Afr Med J. 2012;102:760. 10.7196/SAMJ.5984 22958701

[11] Wiysonge CS Alobwede SM de Marie C Katoto P Kidzeru EB Lumngwena EN COVID-19 vaccine acceptance and hesitancy among healthcare workers in South Africa. Expert Rev Vaccines. 2022;21:549-59. 10.1080/14760584.2022.2023355 34990311

[12] Kahn K Collinson MA Gomez-Olive FX Mokoena O Twine R Mee P Profile: Agincourt Health and Socio-demographic Surveillance System. Int J Epidemiol. 2012;41:988-1001. 10.1093/ije/dys115 22933647 PMC3429877

[13] Brewer NT Chapman GB Rothman AJ Leask J Kempe A Increasing Vaccination: Putting Psychological Science Into Action. Psychol Sci Public Interest. 2017;18:149-207. 10.1177/1529100618760521 29611455

[14] Brewer NT What Works to Increase Vaccination Uptake. Acad Pediatr. 2021;21:S9-16. 10.1016/j.acap.2021.01.017 33958099

[15] Collins K, Holder J. What data shows about vaccine supply and demand in the most vulnerable places. Available: https://www.nytimes.com/interactive/2021/12/09/world/vaccine-inequity-supply.html. Accessed: 22 February 2022.

[16] Kollamparambil U Oyenubi A Nwosu C COVID19 vaccine intentions in South Africa: health communication strategy to address vaccine hesitancy. BMC Public Health. 2021;21:2113. 10.1186/s12889-021-12196-4 34789201 PMC8596859

[17] Burger R, Buttenheim A, English R, Maughan-Brown B. COVID-19 Vaccine Hesitancy in South Africa: Results from NIDS-CRAM Wave 4. Available: https://cramsurvey.org/wp-content/uploads/2021/05/3.-Burger-R.-Buttenheim-A.-English-R.-Maughan-Brown-B.-Kohler-T.-_-Tameris-M.-2021.-COVID-19-vaccine-hesitancy-in-South-Africa-Results-from-NIDS-CRAM-Wave-4.pdf. Accessed: 22 February 2022.

[18] Department of Health Republic of South Africa. COVID-19 Public Dashboard. Available: https://www.covid19sa.org/southafricavaccination Accessed: 22 February 2022.

